# Insights into pyrrolysine function from structures of a trimethylamine methyltransferase and its corrinoid protein complex

**DOI:** 10.1038/s42003-022-04397-3

**Published:** 2023-01-16

**Authors:** Jiaxin Li, Patrick T. Kang, Ruisheng Jiang, Jodie Y. Lee, Jitesh A. Soares, Joseph A. Krzycki, Michael K. Chan

**Affiliations:** 1 School of Life Sciences, and Center of Novel Biomaterials, The Chinese University of Hong Kong, Shatin, Hong Kong, China; 2 Department of Integrative Medical Sciences, College of Medicine, Northeast Ohio Medical University, Rootstown, OH 44272 USA; 3 Ohio State University Biochemistry Program, Columbus, OH 43210 USA; 4 Department of Microbiology, The Ohio State University, Columbus, OH 43210 USA; 5 TechLab, Inc., Blacksburg, VA 24060 USA; 6 Division of Scientific Advancement, American Chemical Society, Washington, DC 20036 USA

**Keywords:** X-ray crystallography, Enzymes

## Abstract

The 22nd genetically encoded amino acid, pyrrolysine, plays a unique role in the key step in the growth of methanogens on mono-, di-, and tri-methylamines by activating the methyl group of these substrates for transfer to a corrinoid cofactor. Previous crystal structures of the *Methanosarcina barkeri* monomethylamine methyltransferase elucidated the structure of pyrrolysine and provide insight into its role in monomethylamine activation. Herein, we report the second structure of a pyrrolysine-containing protein, the *M. barkeri* trimethylamine methyltransferase MttB, and its structure bound to sulfite, a substrate analog of trimethylamine. We also report the structure of MttB in complex with its cognate corrinoid protein MttC, which specifically receives the methyl group from the pyrrolysine-activated trimethylamine substrate during methanogenesis. Together these structures provide key insights into the role of pyrrolysine in methyl group transfer from trimethylamine to the corrinoid cofactor in MttC.

## Abstract (web summary)

Structures of *Methanosarcina barkeri* trimethylamine methyltransferase (MttB) with its substrates reveal the role of pyrrolysine in methyl group transfer from trimethylamine to the corrinoid cofactor in MttC.

## Introduction

The vast majority of methane formed in biological systems is carried out by a special group of Archaea known as methanogens^1–3^. These organisms reduce small one-carbon containing substrates to methane as their primary source of energy. While most methanogens can grow on carbon dioxide, some have evolved alternate pathways^4–7^. For example, some species of methanogens, such as *Methanosarcina barkeri*, have adapted to utilize methylamines (trimethylamine, TMA; dimethylamine, DMA; and monomethylamine, MMA) as substrates for methanogenesis^8–10^.

Methanogens are among the few organisms known to anoxically and completely demethylate TMA. In recent years interest in enzymes producing or consuming TMA, or its precursors, has been growing due to the impact that microbially produced TMA has on human health^11,12^. TMA produced by the gut microbiome is converted in the body to TMA N-oxide (TMAO), a molecule whose presence at elevated concentrations in plasma has been linked to heart disease^13–15^. The demethylation of TMA in the gut by methanogens has been suggested as a possible route to lower the body burden of TMAO^16^. Enzymes for TMA demethylation are encoded in the genomes of intestinal bacteria as well^17,18^. These developments have added additional impetus toward further understanding of the metabolism of TMA by methanogenic Archaea.

The complete demethylation of TMA by methanogens produces the intermediates, DMA and MMA, and requires distinct methyltransferases with specificity for all three methylamine substrates. A substrate:corrinoid methyltransferase catalyzes the demethylation of the specific methylamine, and subsequently transfers this methyl group onto an associated corrinoid protein. A corrinoid:coenzyme M methyltransferase then catalyzes the transfer of the methyl group from the methylated corrinoid to coenzyme M (CoM)^8–10,19,20^. Methyl-CoM is ultimately reduced by methyl-CoM reductase to produce methane^3^.

For TMA:CoM methyl transfer, trimethylamine methyltransferase (MttB) is required to methylate its cognate corrinoid protein, MttC, resulting in DMA^10^. Similarly, dimethylamine methyltransferase (MtbB) and monomethylamine methyltransferase (MtmB) are required to promote methyl group transfer from DMA and MMA to their respective cognate corrinoid proteins, MtbC^9^ and MtmC^8^.

All three corrinoid proteins (MttC, MtbC, and MtmC) are highly homologous and substrates for the same methylcobamide:coenzyme M methyltransferase, MtbA. In contrast, MttB, MtbB, and MtmB, which recognize different methylamine substrates, exhibit no significant sequence homology. One feature that the genes encoding *M. barkeri* MttB, MtbB, and MtmB do share in common, however, is an in-frame amber codon that is translated^21–23^. Instead of acting as a stop, the amber codon signals co-translational insertion of the novel amino acid pyrrolysine by a unique tRNA^Pyl^^23,24^.

The identity of the UAG encoded amino acid, subsequently named pyrrolysine, was determined from the 1.55 Å crystal structure of the *M. barkeri* MtmB which showed density consistent with a lysine residue in amide linkage through its ε-nitrogen to (4R, 5R)-4-methyl-pyrroline-5-carboxylate^23,25,26^ located at the center of the TIM barrel fold comprising each subunit. The characterization of synthetic pyrrolysine^25^ and the biosynthetic pathway for pyrrolysine^27^, as well as the discovery that tRNA^Pyl^ is aminoacylated with pyrrolysine by pyrrolysyl-tRNA synthetase^28,29^ reinforced the conclusion that the pyrrolysine is the 22nd genetically-encoded amino acid found in nature.

Considering the lack of sequence conservation among MttB, MtbB and MtmB extends to the location of the pyrrolysine-encoding amber codon in each methyltransferase^22,26^, the features of pyrrolysine-mediated activation of the DMA and TMA are likely to be quite different from MtmB. To garner additional insight into the role of pyrrolysine in biology, we have been pursuing the structure determinations of the two other known classes of pyrrolysine-containing methyltransferases, MtbB and MttB. Herein, we describe the structure of the *M. barkeri* MttB involved in trimethylamine activation and its complex with its associated corrinoid partner MttC. The MttB structures reveal distinct features in the mode of substrate binding and activation to that of the previously determined MtmB, while the structure of MttB-MttC complex provides insights into the role of pyrrolysine in positioning the trimethylamine substrate for transfer to the corrinoid cofactor.

## Results and discussion

### MttB structure

**Fig. 1:**
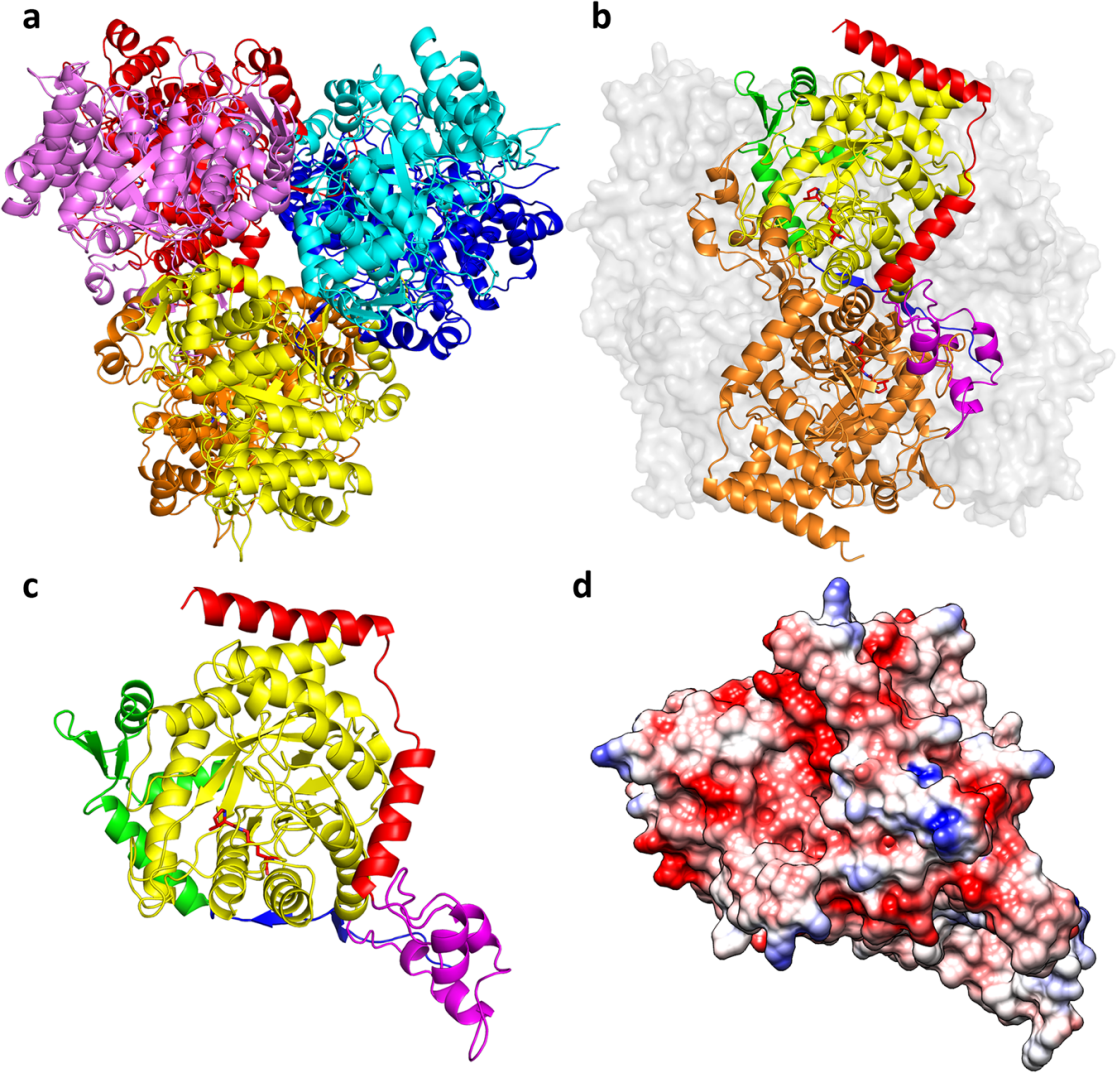
Structure of MttB. Ribbon diagram of MttB hexamer viewed along (**a**) the threefold axis with dimer pairs in more similar colors (yellow, orange; cyan, blue; violet, red); and (**b**) the twofold axis with one subunit colored orange, and the other subunit colored by region: residues 2–22 in blue, 23–81 in green, TIM-barrel 82–395 in yellow, dimerization loop 396–452 in magenta, and 453–495 in red. Pyl334 is shown as red stick. **c** Structure of single subunit in ribbons colored as in (**b**). **d** Surface electrostatic potentials of single subunit with positive in blue (10 kcal/mol·*e*) and negative in red (−10 kcal/mol·*e*) in the same orientation as in (**c**).

The 2.5 Å structure of MttB reveals that the protein exists as a D_3_-symmetric homo-hexamer, although based on the strong protein contacts between pairs of twofold related subunits, the structure is perhaps better considered a trimer-of-dimers (Fig. 1a, b). Similar to other corrinoid-cofactor associated proteins^30,31^, each MttB subunit adopts a TIM-barrel fold, made up by the central residues in the protein sequence (residues 82–395) (Fig. 1c, and Supplementary Fig. 1). The flanking N- and C-terminal regions appear to play major roles in MttB oligomerization (Supplementary Table 1).

A C-terminal loop (residues 396–452) that directly follows the TIM-barrel domain in the sequence forms extensive contacts with a twofold related MttB subunit leading to formation of tightly associated MttB dimers. This C-terminal dimerization loop is primarily random coil but does contain four short α-helices. The remaining part of the C-terminus consists of two long α-helices (α19, residues 453–467 and α20, residues 475–494) that bind to the outside of the TIM-barrel and serve to anchor the dimerization loop.

The dimer is further stabilized by interactions provided by an N-terminal β-strand (β2, residues 17–19). This β-strand lies near the twofold axis between the dimers and forms a two stranded β-sheet with its twofold-related partner. The remaining residues in the N-terminal region appear to stabilize the trimer. The major interaction is provided by an N-terminal α/β subdomain (residues 23–81) which lies near the threefold axis and is positioned on the opposite side of the TIM barrel relative to the dimerization loop. An α-helix (α3, residues 71–80) from this α/β subdomain interacts with the threefold related α helices on the other two dimers forming a triangle. An additional interaction is provided by the most N-terminal β-strand (β1, residues 10–14), which resides near the twofold axis between distinct α_2_ and α’_2_ dimers. Each N-terminal β-strand interacts with the twofold related strand on another α’_2_ dimer resulting in three sets of β-strand interactions between pairs of α_2_ dimers which help to stabilize their trimerization.

**Fig. 2:**
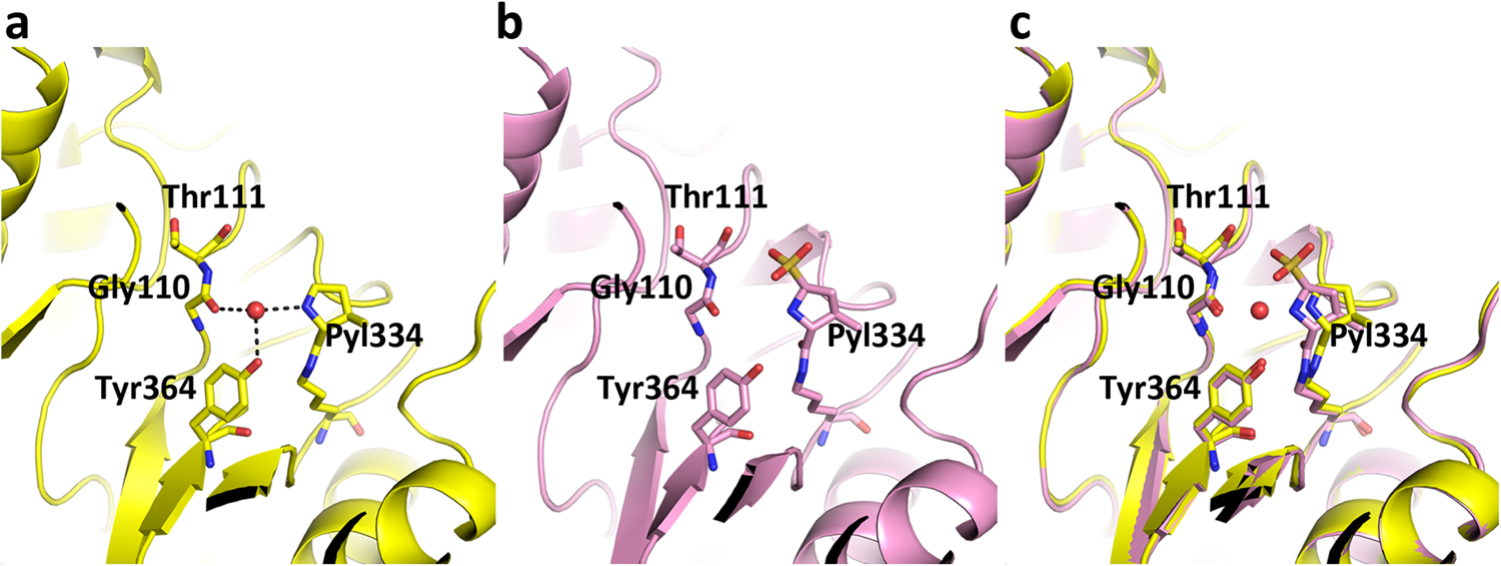
Comparison of the active site in MttB and sulfite-bound MttB. Ribbon diagram of the active site of (**a**) MttB (yellow) and (**b**) sulfite-bound MttB (violet) and (**c**) their superposition. Gly110, Thr111, Pyl334, Tyr364, and the sulfite bound to pyrrolysine are shown in stick with the non-carbon atoms colored in CPK. The water hydrogen bonded to Pyl334 in the MttB structure is shown as a red sphere. This water is absent in the sulfite-bound MttB structure. Hydrogen bonds are shown as dashed lines.

The central question regarding MttB is undeniably the location of the pyrrolysine amino acid. While in the structure of the MtmB, the first structurally characterized pyrrolysine-containing protein, pyrrolysine (Pyl202) is anchored to a β-strand within the TIM-barrel^23^ (Supplementary Fig. 2a), in the MttB structure the pyrrolysine (Pyl334) is located above the β-barrel with its critical pyrroline side chain directed sideways toward the active site cavity (Fig. 1c, Supplementary Fig. 2b). A lone water appears to hydrogen bond the pyrroline imine and this water is in-turn stabilized by hydrogen bonding interactions with the hydroxyl group of Tyr364 and the main chain carbonyl of Gly110 (Fig. 2a, Supplementary Fig. 3). Notably, the orientation of the Gly110 carbonyl is achieved by its participation in a Gly110-Thr111 *cis* peptide-bond. Both Gly110 and Tyr364 are fully (100%) conserved among pyrrolysine-containing trimethylamine methyltransferases (Supplementary Fig. 4).

To evaluate the significance of the Tyr364 phenolic hydrogen bond, a Y364F mutant was prepared. The mutation had only a slight impact on the K_M_ (49 ± 6 µM to 75 ± 11 µM), but led to a significant decrease in the V_max_ (from 48 ± 2 turnovers per min to only 1.7 ± 0.1 turnovers per min, Supplementary Fig. 5), suggesting that Tyr364 plays an important role in the multistep methyltransferase reaction. Our hypothesis is that the hydroxyl group of Tyr364 can stabilize and protonate the water molecule which in turn protonates the imine nitrogen of pyrrolysine thereby promoting nucleophilic attack of the imine carbon by the TMA substrate. Without the Tyr phenolic hydrogen bond, there is less propensity to bind TMA, slowing down the overall reaction process.

### Sulfite addition to pyrrolysine in MttB crystals soaked with dithionite

In our first structure of a pyrrolysine-containing protein, the monomethylamine methyltransferase MtmB, we demonstrated that addition of dithionite to MtmB crystals resulted in addition of a sulfite group to the pyrrolysine imine carbon^25^. This adduct was useful in confirming the presence of the two distinct conformations of pyrroline ring in MtmB, due the relative strong density of the sulfur atom.

We sought to use a similar approach to clarify the details of the conformational states of pyrrolysine in MttB and to provide a structure of this protein bound to a substrate analog. Thus, a MttB crystal was soaked with dithionite and the structure of the resulting MttB-sulfite complex was determined to 3.2 Å resolution. As expected, addition of sulfite to the pyrrolysine imine carbon of MttB was observed with only one strong peak for the sulfite sulfur consistent with a single pyrroline conformation (Fig. 2b, Supplementary Fig. 6b). While the structures of MttB and the MttB-sulfite complex are nearly the same (RMSD = 0.458 and 0.492 Å for the A subunits and overall hexamer, respectively), one significant change was the loss of the water that hydrogen bonds the pyrroline nitrogen in the native MttB structure (Fig. 2).

### Structure of the MttB-MttC complex

**Fig. 3:**
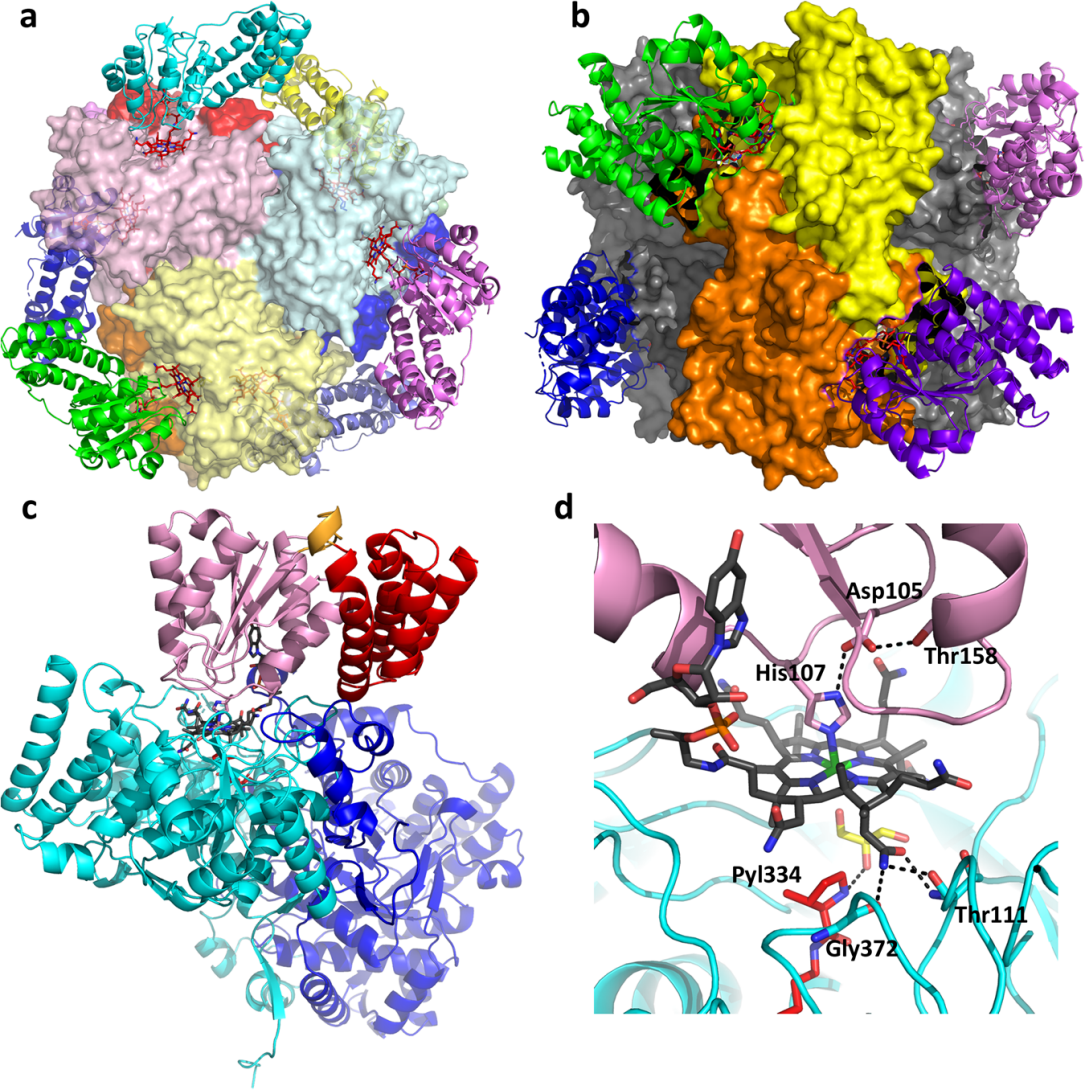
Structure of MttB-MttC complex. Overall structure of MttB-MttC hetero-dodecamer viewed along (**a**) the threefold axis, and (**b**) the twofold axis. MttB subunits colored as in Fig. 1a and shown as surface, corrinoid cofactors shown as red stick and sit in the active site groove of MttB subunits. MttC subunits are shown in ribbon and colored by subunit. **c** Each MttC subunit interacts with a MttB dimer pair in the MttB-MttC complex. MttC colored by region: helical cap domain (residues 2–84) in red, core domain (residues 96–216) in pink, connecting loop (residues 85–95) in orange, and corrinoid cofactor shown as gray stick. One MttB subunit (cyan) interacts with the MttC core domain and corrinoid cofactor, while the second MttB subunit (blue) interacts with both the MttC cap and core. **d** View of active site of MttB-MttC highlighting important residues and their hydrogen-bonding interactions. The nitrogen of His107 is covalently bound to cobalt. The hydrogen-bonded triad (His107, Asp105, and Thr158) in MttC are shown in stick and labeled. The hydrogen bonds between cobamide (gray stick) and MttB (cyan ribbon), as well as between glycerol (yellow stick) and pyrrolysine (red stick) are shown as dashed black lines.

The 2.7 Å resolution structure of MttB-MttC complex was determined from crystals belonging to space group P2_1_ with one full MttB hexamer bound to six MttC monomers with approximate D_3_ symmetry in the asymmetric unit (Fig. 3a, b). The global structure of the MttB subunits in the MttB-MttC complex is nearly unchanged from that of the MttB only structure, with RMSD = 0.815 and 0.801 Å for just the A subunits and entire hexamer, respectively.

Similar to other related corrinoid proteins^32^, the MttC protein in the MttB-MttC complex is composed of two domains, a helical cap domain linked by a flexible loop to an α/β core domain which adopts a Rossmann fold (Fig. 3c). The corrinoid cofactor is positioned at the apex of the core domain with its α-face oriented toward the core. Two loops (residues 101–109 and 138–153) are buttressed against the corrinoid with the side chain of one residue, His107, forming a covalent bond to its cobalt. The electron density of the cofactor is consistent with 5-hydroxybenzimidazolyl cobamide (Supplementary Fig. 7), the predominant form of corrinoid extractable from *Methanosarcina* spp. grown on methanol^33^. The cofactor is most likely in the pentacoordinate Co(II) state, as one nitrogen of His107 binds to the cobalt. The other nitrogen of His107 forms a hydrogen bond with the side chain of Asp105, which in turn is hydrogen-bonded to the side chain of Thr158 (Fig. 3d). This His107-Asp105-Thr158 is part of a conserved ligand triad observed in other corrinoid dependent methyltransferases^34^ (Supplementary Fig. 8) Such ligand triads are key to the dissociation of the histidine ligand during formation of the nucleophilic tetracoordinate Co(I) which accepts a methyl group.

Corrinoid proteins are known to undergo a conformational change upon binding to methyltransferases. In the native state, the helical cap domain blocks the β-face of the corrinoid^32^, but upon methyltransferase binding, the cap domain is shifted to expose the corrinoid cofactor so that it can participate in the methyltransferase reaction^35,36^. A similar positioning of the cap domain away from the corrinoid cofactor is observed in the MttB-MttC complex. This positioning exposes the β-face of the corrinoid cofactor and allows it to bind to the cavity formed by the TIM-barrel of MttB, thereby placing it directly over the pyrrolysine amino acid (Fig. 3c). This binding is stabilized by hydrogen bonds between the corrinoid cofactor and residues Thr111 and Gly372 at the rim of cavity formed by MttB TIM barrel (Fig. 3d). Other than a salt bridge between Glu225 and Arg130, there are no major interactions between the MttC and the particular MttB subunit with which the corrinoid associates (Supplementary Table 2). Instead, it is the corrinoid cofactor, and the dimerization loop (residues 396–452) of the adjacent MttB partner that appear to stabilize the interaction between the primary MttB-MttC catalytic pair (Fig. 3c).

### Structure of the active site in the MttB-MttC complex

**Fig. 4:**
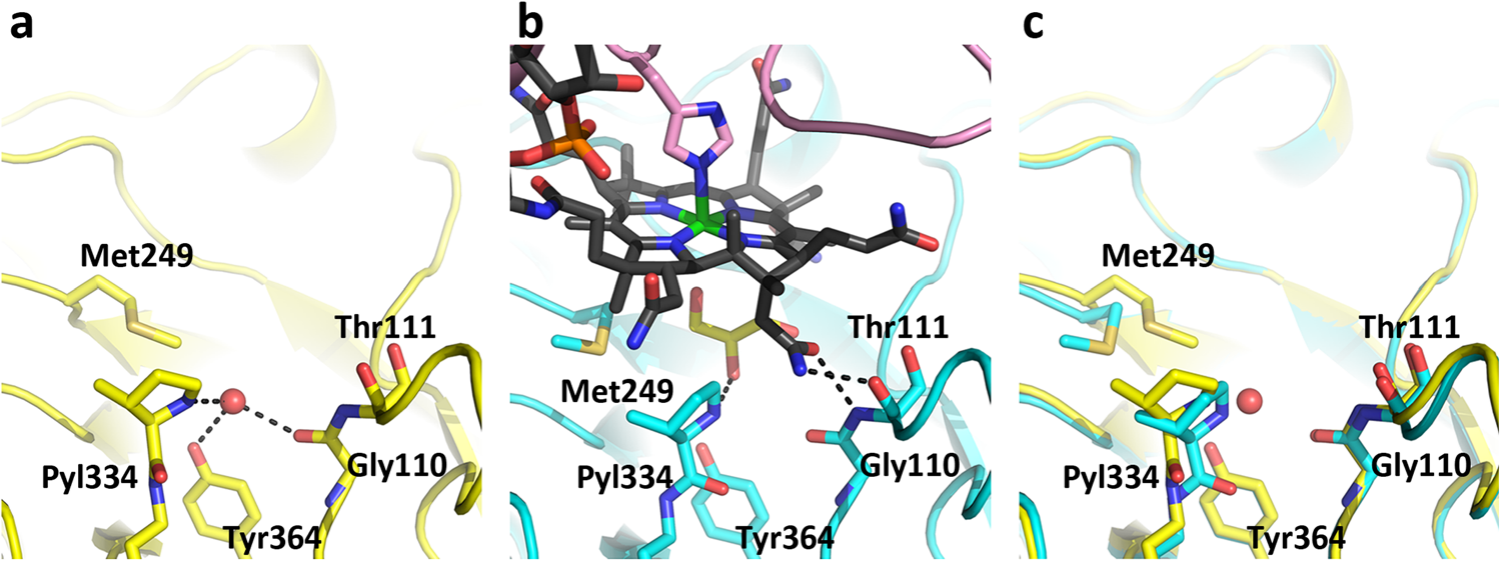
Residues around the pyrrolysine active site change their orientation upon MttC binding. View of the active site of (**a**) MttB (yellow), (**b**) MttB-MttC complex (cyan), and (**c**) their superposition. MttB is shown as ribbon, with Gly110, Thr111, Met249, Pyl334 and Tyr364 shown in stick. Notable changes are (1) the water, shown as a red sphere, which hydrogen bonds to pyrrolysine in isolated MttB is absent in the MttB-MttC complex, (2) the side chain of Met249 moves away from where TMA would be positioned upon addition to pyrrolysine, and (3) a solvent molecule (glycerol) binds to the pocket formed between MttB and the β-face of the corrinoid cofactor.

TMA activation by MttB prior to methyl group transfer to the corrinoid cobalt is thought to occur via formation of a pyrrolysine-bound TMA. In the MttB-MttC complex, there is a cavity between the corrinoid cobalt and pyrrolysine that appears to be occupied by glycerol or water molecule(s) depending on the subunit. Several MttB residues appear to undergo a conformational change upon formation of the MttB-MttC complex (Fig. 4). The most notable of these is Met249, which adopts a conformation that moves its side chain away from the pocket in a fashion that appears suitable for facilitating TMA binding to pyrrolysine and its subsequent positioning near the corrinoid cobalt.

**Fig. 5:**
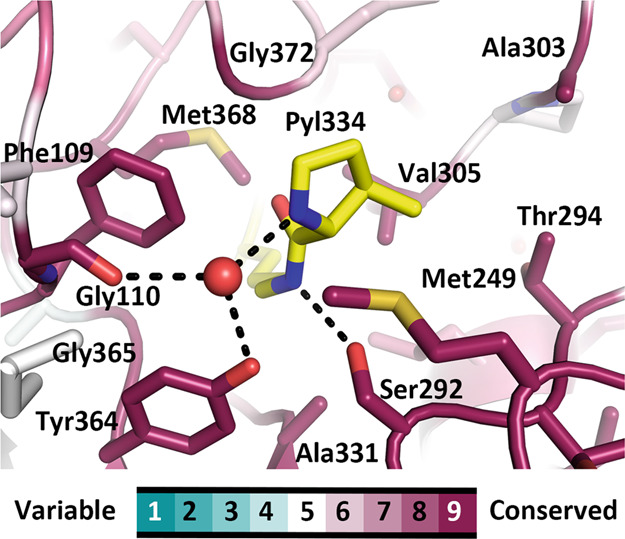
The residues in the MttB active site are highly conserved. Stick diagram of the MttB active site with the pyrrolysine carbons colored yellow, and the carbons of other residues colored by their level of conservation using the ConSurf color scheme (see color bar). The remaining non-carbon atoms are colored in CPK. Fully conserved residues are labeled.

As a further test of the conservation of residues in active site, a multiple sequence alignment of 66 putative pyrrolysine-containing trimethylamine methyltransferases recorded in the UniProt Knowledgebase^37^ was prepared using Clustal Omega^38^ and visualized by Jalview^39^ (Supplementary Fig. 4). This alignment has also been mapped to the structure of MttB (Fig. 5). In keeping with a pivotal role in catalysis, residues around the pyrrolysine appears highly conserved, including the Gly110 and Tyr364 that participating in pyrrolysine activation. Most of the remaining regions that exhibit high conservation on the surface of MttB belong to the regions interact with the C-terminal dimerization loop of its dimer partner, as well as residues in MttB-MttC interface.

### Insights into pyrrolysine-mediated trimethylamine activation and methyl group transfer from the structures of MttB and its complexes

Other than providing information into its three-dimensional fold, perhaps the most important issue with respect to the MttB and MttB-MttC structures is the insight they might provide into the mechanism of pyrrolysine-mediated binding and activation of TMA and how it compares to the mechanism determined for pyrrolysine-mediated activation of MMA from the structure of MtmB. Based on the MttB structures, the mechanism of TMA addition to pyrrolysine to MttB appears to be much simpler than that of MtmB—involving only a single conformation of the pyrroline ring. Here, the pyrroline imine nitrogen is hydrogen bonded to a single water which in turn forms hydrogen bonds to the hydroxyl of Tyr364 and the main chain carbonyl of the Gly110-Thr111 peptide bond. One possibility is that Tyr364-Wat-Pyl334 form a proton shuttle that aids in the generation of the positively charged TMA-pyrrolysine adduct by formation of the tyrosine anion. The lower activity of the Y364F mutant would be consistent with this.

We note that in the MttB-MttC complex (Fig. 4b), the Gly110-Thr111 peptide bond N-H forms a hydrogen bond to one of the amide carbonyl oxygens, O28, of the corrin ring. The roles of the Gly110-Thr111 amide N-H in corrinoid binding in the MttB-MttC complex, and the carbonyl of their peptide bond in hydrogen bonding the water that likely helps to mediate TMA addition in the MttB structure, may indicate a possible linkage between formation of the TMA addition to pyrrolysine, and MttC binding to MttB, though further work would be needed to evaluate this hypothesis.

It should be noted that the water that hydrogen bonds the pyrroline imine nitrogen in the native MttB structure is absent in both the sulfite-bond MttB (Fig. 2b) and the MttB-MttC complex (Fig. 4b). This may suggest that during turnover, addition of TMA to pyrrolysine may precede MttC binding—since nucleophilic addition to pyrrolysine would be slower without a hydrogen bond donor.

In the MttB-MttC complex, the pyrrolysine side chain appears to have no hydrogen bonding interactions with other amino acids in the active site pocket. This is important since the current distance between the imine carbon of pyrrolysine and the corrinoid cobalt is long, ~7 Å (Supplementary Fig. 9a). This is due in part to the fact that, while Met249 has altered its conformation to open the space above the corrinoid cobalt for substrate binding, the pyrroline ring remains on one side of the cavity. It maybe that the TMA-pyrrolysine adduct is more prone to move into the space vacated by Met249, though even then the distance appears too long for transfer to the corrinoid cobalt in its current position.

Molecular modeling of an TMA-pyrrolysine adduct based within binding pocket of the MttB-MttC complex leads us to conclude that the closest the bound TMA can get to the corrinoid cobalt in the MttB-MttC structure is ~4.5 Å (Supplementary Fig. 9b). Given that a Co(III)-methyl distance is around 2.0 Å^32^, a structural change appears to be required in order to bring the corrinoid cofactor and TMA-bound pyrrolysine together so that the methyl group transfer step can take place. We note that a similarly long distance between the site of methyl group activation and the corrinoid cobalt was also observed in the MtaB-MtaC structure^31^. This may indicate that there may be a common mechanism by which this distance is bridged. Notably, during the methyl transfer reaction, the corrinoid is reduced from its inactive Co(II) state observed the MttB-MttC and MtaB-MtaC structures to an active Co(I) state capable of receiving the methyl group. One possible mechanism that would allow the methyltransfer to take place is that upon reduction, the central cobalt ion converts from the pentacoordinate Co(II) state to the tetracoordinate Co(I) state by dissociation of the histidine β-ligand concomitant with His protonation via the ligand triad^40^. Such a transition could result in the corrinoid cofactor moving closer to pyrrolysine, thereby facilitating transfer of the methyl group from the TMA-pyrrolysine adduct of MttB to the MttC cofactor.

### Comparison of MttB with MtmB

Given their similar role in methylamine activation, a structural comparison of MttB and MtmB is interesting for evaluation of similarities and differences. Both proteins adopt the TIM-barrel fold and are homohexamers with D_3_ symmetry—though the relative positions of the subunits are different. In terms of the individual subunits, a comparison of the two proteins reveals that MttB is more complex (Supplementary Fig. 2). MtmB consists of a classical TIM-barrel protein with a central β-barrel and surrounding α-helices. MttB, however, has additional N-terminal and C-terminal sequences that extend beyond the TIM-barrel region (Fig. 1c). As mentioned previously, these extra regions in MttB are responsible for stabilizing the MttB packing, and the dimerization loop not only stabilizes dimer formation but also contributes to the interaction with MttC. These differences may play a role in promoting the specificity of methyltransferases toward their respective partner corrinoid protein.

It should be noted that while the pyrrolysine residue in both proteins point toward the center of the TIM-barrel, this residue originates from a completely different sides of the barrel in these two proteins (Supplementary Fig. 2)—an observation that may have potential implications to the origins and evolution of mono-, di-, and tri-methylamine methyltransferases. While it would be conceivable that a single methylamine methyltransferase evolved to utilize pyrrolysine and then other methyltransferases evolved from this parent, the lack of discernable sequence similarity, and the fact that the pyrrolysine in the three distinct methyltransferases is located in different regions of the sequence and fold, suggest that pyrrolysine may have evolved for use in one methylamine methyltransferase, and then was incorporated into two other evolutionarily distinct TIM-barrel proteins resulting in methyltransferases with different methylamine substrate specificities.

### Other structural homologs of MttB

Use of the program DALI^41^ to search for structural homologs of MttB led to the identification of members from several other protein families (Supplementary Table 3). The highest scoring hits include (1) the glycine betaine methyltransferase (MtgB^42^, PDB ID: 2QNE) which also has high sequence similarity and was used for the molecular replacement in MttB structure determination; (2) the monomethylamine methyltransferase (MtmB^23^, PDB ID: 1NTH) described above; (3) the delta and gamma subunits of the acetyl-CoA decarbonylase/synthase complex (ACDS^43^, PDB ID: 4C1N), also known as the small and large subunits of corrinoid iron-sulfur protein (CoFeSP^44^, PDB ID: 4DJD), as well as the methyltransferase (PDB ID:2YCJ) which catalyzes methyl transfer from methyltetrahydrofolate to CoFeSP in the Wood-Ljungdahl carbon fixation pathway for acetyl-CoA synthesis using only CO_2_ as carbon source^45,46^; and (4) 5-methyltetrahydrofolate S-homocysteine methyltransferase/methionine synthase (MetH^30^, PDB ID: 1Q8J), a multimodular enzyme which is the only cobalamin-dependent methyltransferase in humans and other mammals that pass a methyl group from methyltetrahydrofolate to homocysteine and produce methionine. Notably, each of these proteins contains a TIM barrel fold with an active site involved in activating a substrate to promote transfer of a methyl group to a corrinoid cofactor in an associated corrinoid-containing protein. Presumably, the structural similarity of MttB with these proteins is a reflection of their similar functional role.

### Insights from comparison of the MttB-MttC and MtaB-MtaC complexes

Given that corrinoid proteins that accept the methyl group from methylotrophic substrates exhibit high sequence similarity, one notable feature is the specificity of methyltransferases for transferring activated methyl groups to their cognate corrinoid protein. For example, MtbB can transfer a methyl group from DMA to MtbC, but not to MtmC^9^. To gain insight into the origins of this specificity, the MttB-MttC complex was compared with the previously determined methanol methyltransferase:corrinoid protein (MtaB-MtaC) complex^31^ (PDB ID: 2I2X) with the goal of elucidating the features that lead to their specific interactions.

As mentioned previously, the interface between the cap domain along with loops of core domain of MttC and dimerization loop of MttB provide the primary interactions that stabilize the MttB-MttC complex. Importantly, this interaction is not with the MttB subunit the corrinoid cofactor binds to, but to the C-terminal dimerization loop of its dimer-associated MttB partner (Fig. 3c). Notably, this design provides additional specificity beyond the interaction itself, as it also requires that the MttB has the proper oligomeric arrangement for positioning of this interaction as well.

The methanol-activating MtaB-MtaC complex from *M. barkeri* consists similarly of a methyltransferase MtaB with a TIM-barrel fold, and a corrinoid protein MtaC with both sequence (37% identity) and structural similarity (C_α_ RMSD = 1.258 Å) to MttC. Analysis of the two structures and sequences suggests that the major difference is the presence of an additional N-terminal domain on MtaC that is absent in MttC. In the structure of the MtaB-MtaC complex, this additional N-terminal domain fits in a groove between two MtaB subunits in a fashion that appears to stabilize complex formation (Supplementary Fig. 10). Notably, sequence alignments of MtaC with the corrinoid proteins involved in demethylating DMA or MMA—MtbC and MtmC respectively, indicate all three methylamine corrinoid proteins lack this additional N-terminal domain^47^ (Supplementary Figs. 8 and 11). Thus, for these proteins there is likely a distinct mechanism of corrinoid specificity. Given the fact that all of the methanogenic methyltransferases characterized to date are oligomeric, this corrinoid specificity could be achieved via interactions to neighboring methyltransferase subunits as observed in the MttB-MttC complex.

## Conclusions

In summary, we have determined the structures of native MttB, and its complexes with a substrate analog, sulfite, and its physiological relevant corrinoid protein, MttC. These structures have provided new insights into the mechanism of activation of pyrrolysine that is distinct from the previously determined MtmB structure, and details of the key steps in the pyrrolysine-mediated transfer of methyl groups from methylamine substrates to the cobalamin cobalt in corrinoid proteins. Finally, we have provided a structural basis for understanding function of the only enzyme documented to anoxically detoxify the proatherosclerotic microbial metabolite TMA by demethylation.

## Methods

### Protein expression and purification

The *mttB1* gene was amplified from *M. barkeri* MS genomic DNA. PCR was carried out with *Ex Taq* DNA polymerase (Takara Mirus Bio, Madison, WI) using 5′-CATATGGCAAAAAATAAT-3′ and 5′-CCGCGGTTATTATTAGTGGTGGTGGTGGTGGTGTCTCCCATGCCTCTGAAGGCTTTGTCAGCCTT-3′ as primers to add a 5′ NdeI site and a 3′ SacII site to the gene, and to include a GGHHHHHH sequence at the C-terminus of the translated product. The PCR product was cloned into pTopo 2.1 (Invitrogen, Carlsbad, CA). Subsequently, the gene was excised with NdeI and SacII and the fragment used to replace the hexahistidine-tagged *mtmB1* gene in pDL03^48^ to create plasmid pTBCH. The upstream p*mcr* promoter and the *mttB1* gene were then excised from pTBCH using SphI and inserted into pWM311^49^ to form a plasmid pTB311. The *mttC* gene was cloned using similar strategy. Primers 5′-CATATGCAAACAAAGAAGAAATC-3′ and 5′-CCGCGGTTAGTGGTGGTGGT-GGTGGTGTCCTCCGACATTCAGGGCTGCTTTTACC-3′ were used to amplify the *mttC* gene. Then the *mttC* gene was inserted into vector pWM311 to form a plasmid pTC311.

Plasmids containing *mttB* or *mttC* with a C-terminal hexahistidine tag were transformed into *Methanosarcina acetivorans* C2A cells by use of anaerobic polyethylene glycol 4000^50^, and the resulting cells were grown at 37 °C on high salt medium^51^ supplemented with 80 mM methanol, 40 mM acetate, and 2 µg/mL puromycin under a 80% N_2_/20% CO_2_ gas mixture for 4 days. The cells were then lysed by French Press in 20 mM sodium phosphate buffer pH 7.4, 500 mM NaCl, 10 mM imidazole, and loaded onto a 1 mL HiTrap HP column (GE Healthcare BioSciences, Piscataway, NJ). The bound proteins were eluted with a gradient of 10–500 mM imidazole in 500 mM NaCl and 20 mM potassium phosphate buffer pH 7.4. The MttB-His_6_ and MttC-His_6_ proteins eluted around 200–250 mM imidazole. Fractions containing MttC were pooled and exchanged into a 40 mM Tris buffer pH 7.0 with 50 mM NaCl and further purified using Mono-Q HR 10/10 column (Amersham Biosciences, Piscataway, N.J.) and eluted with around 200–250 mM NaCl.

### MttB Tyr364 substitution with Phe

The *mttB1* gene was subjected to site-directed mutagenesis employing primers 5′-GCCGGTGCCAACACCATCTTCGGAGCTGGAATGCTTGAGC-3′ and 5′-GCTC-AAGCATTCCAGCTCCGAAGATGGTGTTGGCACCGGC-3′, to create plasmid pTB311Y364F. MttB-Y364F was then expressed and purified as described above for wild type MttB.

### Dicyano assay and molar extinction coefficients determination of MttC

Dicyano assays were used to determine the molar stoichiometry of the corrinoid cofactor in recombinant MttC. A 150 µL solution containing 50 mM CHES buffer, pH 10, 2% SDS, 30 µM MttC was incubated in a 1.5 mL microcentrifuge tube at 65 °C for 30 min. The tube was then centrifuged at 10,000 *rpm* for 10 min at 4 °C. The absorbance of the supernatant was then taken at 368 nm. Subsequently, 2.5 µL of 80 mM KCN was added to the supernatant and after 20 min incubation at room temperature, a second absorbance was taken at 368 nm. The concentration of dicyanocobalamin was determined by applying the experimentally determined extinction coefficient of 12,500 M^−1^ cm^−1^ at 368 nm. Recombinant MttC preparations bound 0.94 ± 0.09 corrinoid cofactor (mol/mol), which is similar to that previously determined for native MttC^10^.

### Spectrophotometric assay of MttB activity

To determine whether recombinant MttB and MttC were active, spectrophotometric assays were conducted with RamA, an iron-sulfur protein isolated from *M. barkeri* that is required for in vitro ATP-dependent reductive activation of methylamine:CoM methyl transfer from all three methylamines^52^. Prior to analysis, MttC was reduced from Co(II) to the Co(I) state by pre-incubation with RamA, Ti(III)citrate, and ATP. Reduction to Co(I) was confirmed by the appearance of the characteristic peak at 386 nm. The methylation of Co(I)-MttC with TMA was initiated by the addition of MttB and followed by the increase in absorbance at 534 nm due to formation of methyl-Co(III)-MttC. Methylation of Co(I)-MttC by MttB with TMA was monitored in stoppered masked quartz sub-micro cuvettes (Starna Cells, Inc.) of 1 cm pathlength. The complete reaction mixture (100 µL) contained 2 mM Ti(III)-citrate, 2.5 mM ATP, 2.5 mM MgCl_2_, 1–5 µM RamA, 30 µM MttC, 0.1 to 1 µM MttB and TMA in 22 mM phosphate buffer, pH 7.2. Reaction mixtures were assembled in an anaerobic chamber (Coy Laboratories, Inc.) with an atmosphere of 2% H_2_ in N_2_. Cuvettes were stoppered and removed from the chamber and subsequent anaerobic additions of protein were made by Hamilton syringe. Reactions took place under dim red light at 37 °C and complete spectra were monitored at 1 s intervals using an HP 8453 diode-array spectrophotometer. Initial reaction rates were determined with a range of substrate concentrations (1–200 µM).

### Statistics and reproducibility

Statistical analysis was performed using GraphPad Prism 6 (GraphPad) using Michaelis-Menten model to determine kinetic parameters. Data were presented as the mean ± SD and individual data points of triplicate assays.

### Crystallization and data collection of MttB and sulfite-bound MttB

The crystallization of *M. barkeri* MttB was performed at 10 °C using the sitting-drop vapor-diffusion method. Initial crystals were obtained from condition 49 of the MPD Suite (QIAGEN). The final optimized crystals used for data collection were grown from hanging drops containing 1 µL protein (8 mg/mL MttB in 20 mM potassium phosphate buffer pH 7.4, 500 mM NaCl, 240 mM imidazole, 20% glycerol) and 1 µL reservoir solutions (4% MPD, 0.1 M citric acid pH 4.5). The dithionite-bound crystals were obtained by transferring the MttB crystals to reservoir solution containing saturated sodium dithionite for 4 min. Both crystals were directly transferred to the reservoir solution containing 30% glycerol prior to being flash cooled in liquid N_2_. The diffraction data of native MttB were collected on beamline 13B at a wavelength of 0.99984 Å using an ADSC Quantum 315 R detector at the National Synchrotron Radiation Resource (NSRRC) in Hsinchu, Taiwan, with the supporting software Blu-Ice and the automated sample mounting system^53,54^. Data processing and scaling were performed with HKL2000^55^. For the sulfite-bound MttB crystal, diffraction data were collected using an in-house rotating anode X-ray generator (Rigaku FRE+) at a wavelength of 1.54187 Å and a RAXIS IV imaging plate detector, and processed with iMosflm^56^ and SCALA^57^ from the CCP4i suite^58^.

### Phase determination and refinement of MttB and sulfite-bound MttB

The native MttB structure was determined by molecular replacement with the program PHASER^59^ running under the CCP4i interface^60^ using the structure of the glycine betaine methyltransferase MtgB^42^ (PDB ID: 2QNE) as the search model. According to the BLASTP 2.12.0 program^61,62^, the two protein sequences have 31% identity and 49% similarity. Iterative cycles of model building and refinement with the programs Coot^63^ and CNS^64^, respectively, were carried out to improve the model. The quality of the final model was evaluated using the program PROCHECK^65^ and summarized in Table 1. In the 2.5 Å resolution MttB structure, 95.7% of the residues were in the favored regions, 4.1% in the allowed regions, and 0.3% in the outlier regions of the Ramachandran plot. The surface electrostatic potentials of MttB were calculated according to Coulomb’s law using Chimera^66^.

**Table 1:** Data collection and refinement statistics.

	Native MttB	sulfite-bound MttB	MttB-MttC
Data collection*			
Wavelength (Å)	0.99984	1.54187	0.99984
Space group	P 3 2 21	P 3 2 21	P 1 21 1
Cell dimensions			
* a*, *b*, *c* (Å)	175.5, 175.5, 301.0	174.8, 174.8, 300.1	120.4, 188.1, 124.0
α, β, γ (°)	90.0 90.0 120.0	90.0 90.0 120.0	90.0, 119.3, 90.0
Resolution (Å)	20 (2.5)	20 (3.2)	20 (2.7)
*R*_merge_	0.091 (0.886)	0.196 (0.421)	0.078 (0.574)
CC1/2	0.993 (0.585)	0.949 (0.821)	0.986 (0.729)
*Ι* / σ_*Ι*_	18.0 (1.9)	2.6 (1.8)	14.5 (2.1)
Completeness (%)	92.1 (93.8)	85.4 (90.9)	99.8 (100)
Redundancy	5.4 (5.0)	2.7 (3.2)	3.5 (3.4)
*Refinement*			
Resolution (Å)	2.5	3.2	2.7
No. reflections	157,837	74,084	124,909
*R*_work_/*R*_free_	17.45/21.77	16.65/24.22	19.20/23.86
No. atoms			
Protein	23,122	22,588	32,514
Ligand/ion	72	144	564
Water	574	165	373
*B*-factors(Å^2^)			
Protein	47.677	53.158	47.119
Ligand/ion	72.910	67.913	55.617
Water	40.755	28.767	26.810
R.m.s. deviations			
Bond lengths (Å)	0.007	0.007	0.007
Bond angles (°)	1.256	1.278	1.251

^*^Values in parentheses are for highest-resolution shell.

The 3.2 Å sulfite-bound MttB structure was determined by molecular replacement based on the native MttB structure. Based on PROCHECK, 89.3% of the residues were in the favored regions, 9.9% in the allowed regions, and 0.8% in the outlier regions of the Ramachandran plot. The coordinates and structure factors of the native and sulfite-bound MttB structures have been deposited in the Protein Data Bank (PDB ID: 7XCL and 7XCM, respectively). All molecular graphics were prepared with PyMOL^67^. Sequence alignment was performed using Clustal Omega^38,68^ and visualized by Jalview^39^.

### Crystallization and data collection of the MttB-MttC complex

Crystals of the MttB-MttC complex were obtained by vapor diffusion at 10 °C from hanging-drops comprised of 0.5 µL MttB (3 mg/mL in 16 mM potassium phosphate buffer pH 7.4, 400 mM NaCl, 160 mM imidazole, 20% glycerol and 1.6 mM DTT), 0.5 µL MttC (10 mg/mL in 40 mM Tris buffer pH 7, 200 mM NaCl and 20% glycerol) and 1 µL reservoir solution (18% PEG8000, 0.1 M citric acid pH 5, and 1 M LiCl) optimized from condition 18 from Wizard Classic 3 (Rigaku). The resulting pink crystals were then soaked with reservoir solution containing 12.5 mM N,N-dimethyl-hydroxylamine for 4 days, before being directly flash cooled in liquid N_2_. The diffraction data for the MttB-MttC complex were collected on microfocus beamline 05 A at a wavelength of 0.99984 Å using RAYONIX MX-300 HS detector at NSRRC. Data processing and scaling were performed with HKL2000^55^.

### Phasing and refinement of the MttB-MttC complex

The MttB component was determined using PHASER^59^ with aforementioned MttB structure (PDB ID: 7XCL). The initial model of MttC was generated using CHAINSAW^69^ based on the structure of the related MtmC (PDB ID: 3EZX; 41% identity, 63% similarity according to BLASTP 2.12.0^61,62^), and was manually adjusted to fit the electron density with the program Coot^63^. Model building and refinement of the complex were carried out to 2.7 Å resolution using the programs Coot^63^ and CNS^64^ and the quality of the final model was evaluated using the program PROCHECK^65^. It shows 93.6% of the residues were in the favored regions, 5.6% in the allowed regions, and 0.7% in the outlier regions of the Ramachandran plot. The coordinate and structure factor have been deposited in the Protein Data Bank (PDB ID: 7LCN).

### Molecular modeling of pyrrolysine bound to TMA in the MttB-MttC complex

To determine whether pyrrolysine could position the TMA in close enough proximity to the corrinoid to facilitate the methyl transfer reaction in the structurally determined MttB-MttC complex, the chi angles of pyrrolysine were adjusted in Coot^63^ so that a TMA bound to pyrrolysine would be positioned above the corrinoid such that the Co(corrinoid)-Me(TMA)-N(TMA) angle was linear—the orientation presumed to be optimal for the methyl transfer reaction.

### Reporting summary

Further information on research design is available in the Nature Portfolio Reporting Summary linked to this article.

## Supplementary information

Supplementary Information

Reporting Summary

## Data Availability

The coordinates and structure factors of of the native MttB, sulfite-bound MttB, and MttB-MttC complex structures have been deposited in the Protein Data Bank (PDB ID: 7LCL, 7LCM and 7LCN, respectivly).
