# Supplementary material for: Insights into pyrrolysine function from structures of a trimethylamine methyltransferase and its corrinoid protein complex
Source: Commun Biol. 2023 Jan 16;6:54. doi: 10.1038/s42003-022-04397-3 (PMC9842639; doi:10.1038/s42003-022-04397-3)
Supplement: Supplementary file 1 — Supplementary Information [file 42003_2022_4397_MOESM1_ESM.pdf]

Supplementary Information

# Insights into pyrrolysine function from structures of a trimethylamine methyltransferase and its corrinoid protein complex

Jiaxin Li, Patrick T. Kang, Ruisheng Jiang, Jodie Y. Lee, Jitesh A. Soares, Joseph A. Krzycki*, Michael K. Chan*

*To whom correspondence should be addressed.

Dr. Michael K. Chan

Office: 852-39431487, Fax: 852-26035646, Email: michaelkchan88@cuhk.edu.hk

Dr. Joseph A. Krzycki

Office: 614-292-1578, Fax: 614-292-8120, Email: krzycki.1@osu.edu

**This document includes:**

Supplementary Table 1. Hydrogen bonded and salt bridged residues in the dimerization and trimerization interfaces in MttB.

Supplementary Table 2. Hydrogen bonded and salt bridged residues between MttB and MttC.

Supplementary Table 3. First 25 results of a DALI search in PDB for structural similarities among MttB homologous proteins.

Supplementary Figure 1. Secondary structure assignment of MttB.

Supplementary Figure 2. Structural alignment of MtmB (PDB ID: 1NTH) and MttB subunits and the relative positions of their pyrrolysine residues.

Supplementary Figure 3. View of the $2F_O$-$F_C$ electron density map of hydrogen-bonding residues near Pyl334.

Supplementary Figure 4. Sequence conservation in pyrrolysine-containing trimethylamine methyltransferases.

Supplementary Figure 5. Substitution of the Tyr364 in MttB with phenylalanine results in a less active enzyme with higher $K_M$.

Supplementary Figure 6. View of the electron density map of residues around pyrrolysine in MttB and sulfite-bound MttB.

Supplementary Figure 7. View of the $2F_O$-$F_C$ electron density map of the corrinoid cofactor and its bound histidine in the MttB-MttC structure.

Supplementary Figure 8. Sequence alignment of corrinoid protein MtmC, MtbC, MttC and MtaC.

Supplementary Figure 9. The distance between pyrrolysine and the pentacoordinate His-bound corrinoid cofactor in the MttB-MttC complex is not suitable for methyl transfer reaction.

Supplementary Figure 10. Structure of MtaB-MtaC tetramer (PDB ID: 2I2X).

Supplementary Figure 11. Superposition of $C_\alpha$ traces of MttC and MtaC.

**Supplementary Table 1. Hydrogen bonded and salt bridged residues in the dimerization and trimerization interfaces in MttB.**

| Region | Residue in one chain | Residue in its dimer partner |
|---|---|---|
| N-terminal (Residue 2-22) | Asn11 | Thr22 |
| | Asn11 | Asp24 |
| | Leu13 | Glu25 |
| | Glu17 | Asn19 |
| | Asn19 | Glu17 |
| | Thr22 | Asn11 |
| N-terminal helical (Residue 23-81) | Asp24 | Asn11 |
| | Glu25 | Leu13 |
| TIM barrel (Residue 82-395) | Pro223 | Gly415 |
| | Gly254 | Phe418 |
| | Gly262 | Thr403 |
| | Asp297 | Arg439 |
| | Lys299 | Asp429 |
| | Thr302 | Arg439 |
| | Glu309 | Ser432 |
| | Lys338 | Asp438 |
| | Lys338 | His440 |
| | Asp341 | Leu453 |
| | Gln343 | Asp438 |
| | His346 | Thr349 |
| | Thr349 | His346 |
| C-terminal dimerization loop (Residue 397-452) | Thr403 | Gly262 |
| | Gly415 | Pro223 |
| | Phe418 | Gly254 |
| | Asp429 | Lys299 |
| | Ser432 | Glu309 |
| | Asp438 | Lys338 |
| | Asp438 | Gln343 |
| | Arg439 | Asp297 |
| | Arg439 | Thr302 |
| | His440 | Lys338 |
| C-terminal helix (residue 453-495) | Leu453 | Asp341 |
| | **Residue in one chain** | **Residue in its trimer partner** |
| N-terminal helical (Residue 23-81) | Glu35 | Arg84 |
| | Glu71 | Ser83 |
| | Glu71 | Arg84 |
| | Arg75 | Leu78 |
| | Arg75 | Gln79 |
| | Arg75 | Ala81 |

**Supplementary Table 2. Hydrogen bonded and salt bridged residues between MttB and MttC.**

| | **Residue in MttB** | **Atom in MttC cofactor** |
|---|---|---|
| Hydrogen bond | Thr111 | O28 |
| | Asp184 | N40 |
| | Cys219 | O39 |
| | Leu371 | N62 |
| | Gly372 | N29 |
| Salt bridge | **Residue in MttB** | **Residue in MttC** |
| | Glu225 | Arg130 |

**Supplementary Table 3. First 25 results of a DALI search in PDB for structural similarities among MttB homologous proteins.**

| | PDB-Chain | Z | rmsd | Equivalent residues | Total residues | % Identity | Description |
|---|---|---|---|---|---|---|---|
| 1 | 2QNE-B | 55.1 | 1.7 | 465 | 475 | 29 | Glycine betaine methyltransferase (MtgB) |
| 2 | 2QNE-A | 54.4 | 1.7 | 466 | 476 | 29 | Glycine betaine methyltransferase (MtgB) |
| 3 | 4YYC-A | 50.5 | 1.9 | 458 | 479 | 26 | Putative trimethylamine methyltransferase* |
| 4 | 1NTH-A | 25.3 | 2.7 | 325 | 457 | 12 | Monomethylamine methyltransferase (MtmB) |
| 5 | 1L2Q-A | 25.2 | 2.8 | 325 | 457 | 12 | Monomethylamine methyltransferase (MtmB) |
| 6 | 1TV3-A | 25.2 | 2.7 | 325 | 457 | 12 | Monomethylamine methyltransferase (MtmB) |
| 7 | 1TV4-A | 25.2 | 2.8 | 325 | 457 | 12 | Monomethylamine methyltransferase (MtmB) |
| 8 | 1TV2-A | 25.1 | 2.8 | 325 | 457 | 12 | Monomethylamine methyltransferase (MtmB) |
| 9 | 4C1N-B | 18.4 | 3.8 | 244 | 309 | 10 | Acetyl-CoA decarbonylase/synthase complex δ subunit |
| 10 | 4C1N-F | 18.4 | 3.8 | 244 | 309 | 10 | Acetyl-CoA decarbonylase/synthase complex δ subunit |
| 11 | 4C1N-D | 18.3 | 3.7 | 244 | 309 | 10 | Acetyl-CoA decarbonylase/synthase complex δ subunit |
| 12 | 2H9A-B | 18.3 | 3.4 | 240 | 307 | 10 | Acetyl-CoA decarbonylase/synthase complex δ subunit |
| 13 | 4C1N-H | 18.2 | 3.7 | 244 | 309 | 10 | Acetyl-CoA decarbonylase/synthase complex δ subunit |
| 14 | 2YCL-B | 18.1 | 3.7 | 244 | 309 | 10 | Acetyl-CoA decarbonylase/synthase complex δ subunit |
| 15 | 2H9A-A | 17.9 | 3.7 | 242 | 383 | 9 | Acetyl-CoA decarbonylase/synthase complex γ subunit |
| 16 | 4DJD-D | 17.8 | 3.8 | 248 | 323 | 10 | 5-methyltetrahydrofolate corrinoid/ iron sulfur protein (CFeSP) small subunit |
| 17 | 1Q8J-A | 17.8 | 3.2 | 237 | 561 | 8 | 5-methyltetrahydrofolate S-homocysteine methyltransferase/cobalamin-dependent methionine synthase (MetH) |
| 18 | 4C1N-A | 17.8 | 3.6 | 245 | 442 | 9 | Acetyl-CoA decarbonylase/synthase complex γ subunit |
| 19 | 1Q85 -A | 17.7 | 3.2 | 238 | 559 | 8 | Cobalamin-dependent methionine synthase (MetH) |
| 20 | 1Q7Z-A | 17.7 | 3.2 | 236 | 559 | 8 | Cobalamin-dependent methionine synthase (MetH) |
| 21 | 4DJE -D | 17.7 | 3.7 | 249 | 323 | 10 | 5-methyltetrahydrofolate corrinoid/ iron sulfur protein small subunit |
| 22 | 4DJF -D | 17.7 | 3.7 | 248 | 323 | 10 | 5-methyltetrahydrofolate corrinoid/ iron sulfur protein small subunit |
| 23 | 4C1N-E | 17.7 | 3.1 | 241 | 442 | 9 | Acetyl-CoA decarbonylase/synthase complex γ subunit |
| 24 | 4DJD-F | 17.7 | 3.7 | 247 | 323 | 10 | 5-methyltetrahydrofolate corrinoid/ iron sulfur protein small subunit |
| 25 | 4C1N-C | 17.7 | 3.7 | 246 | 442 | 9 | Acetyl-CoA decarbonylase/synthase complex γ subunit |

* 4YYC shares 45% (212/475) sequence identity with 2QNE.

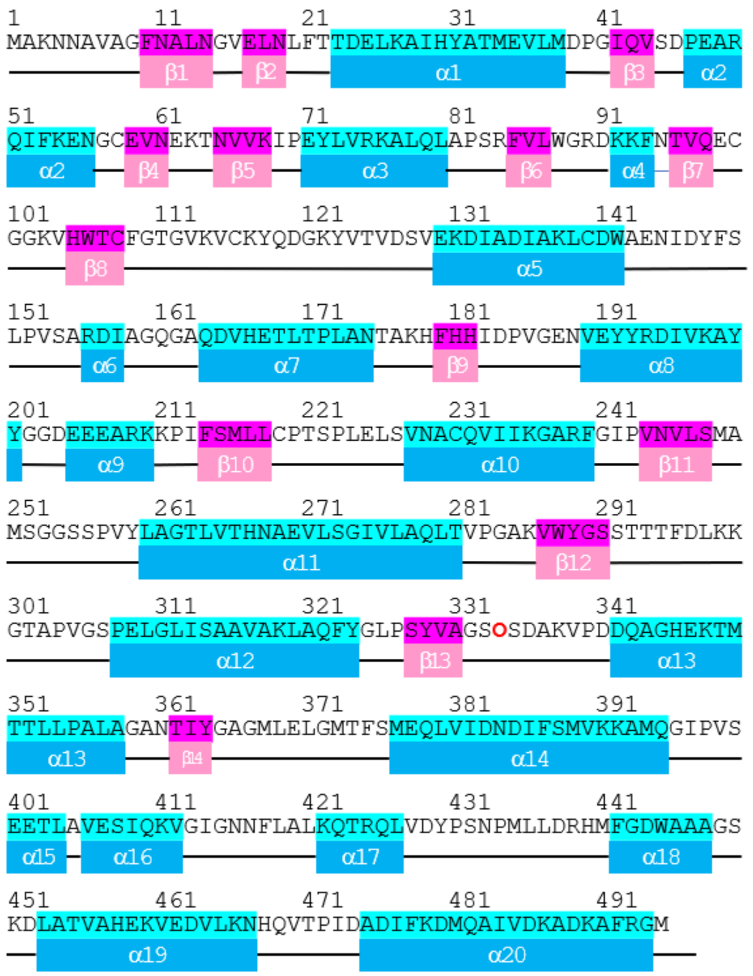

**Supplementary Figure 1. Secondary structure assignment of MttB.** The secondary structural elements are labeled according to their occurrence in sequence with α-helices and β-strands determined in PyMOL, and visualized as a cyan or pink box respectively. α-helix(residues): α1(23-38); α2(47-56); α3(71-80); α4(92-94); α5(130-142); α6(156-158); α7(164-175); α8(190-201); α9(205-210); α10(228-240); α11(260-281); α12(308-324); α13(342-358); α14(377-395); α15(401-404); α16(406-412); α17(422-427); α18(442-448); α19(453-467); α20(475-494); β-strand(residues): β1(10-14); β2(17-19); β3(42-44); β4(59-61); β5(65-68); β6(85-87); β7(96-98); β8(105-108); β9(180-182); β10(214-218); β11(244-248); β12(287-291); β13(328-331); β14(362-366).

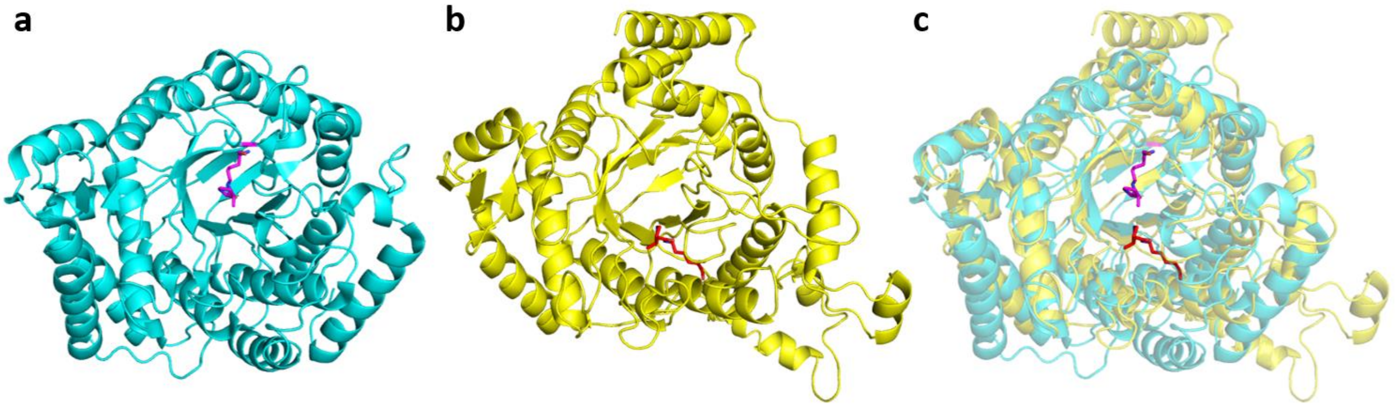


**Supplementary Figure 2. Structural alignment of MtmB (PDB ID: 1NTH) and MttB subunits and the relative positions of their pyrrolysine residues.** Top view of (**a**) MtmB, (**b**) MttB and (**c**) their superposition. MtmB in cyan ribbon with the Pyl202 (in main conformation, occupancy 0.85) as magenta stick. MttB in yellow ribbon with the Pyl334 shown in red stick.

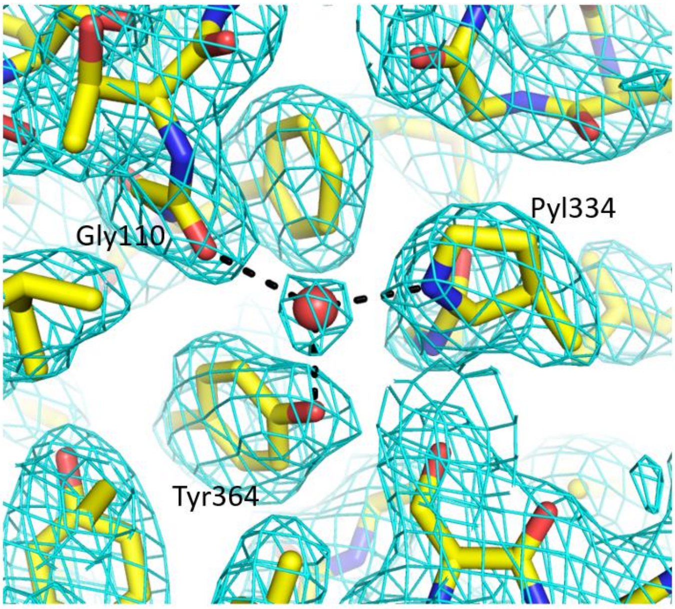


**Supplementary Figure 3. View of the 2F$_O$-F$_C$ electron density map of hydrogen-bonding residues near Pyl334.** MttB shown as yellow stick, and the water hydrogen binding to Gly110, Pyl334 and Tyr364 shown as a red sphere. The simulated annealing omit composite map was calculated using the program CNS and displayed at a sigma level of 1.2 (cyan mesh). All subunits in the native MttB structure possess a water molecule hydrogen bond to Gly110, Pyl334 and Tyr364.

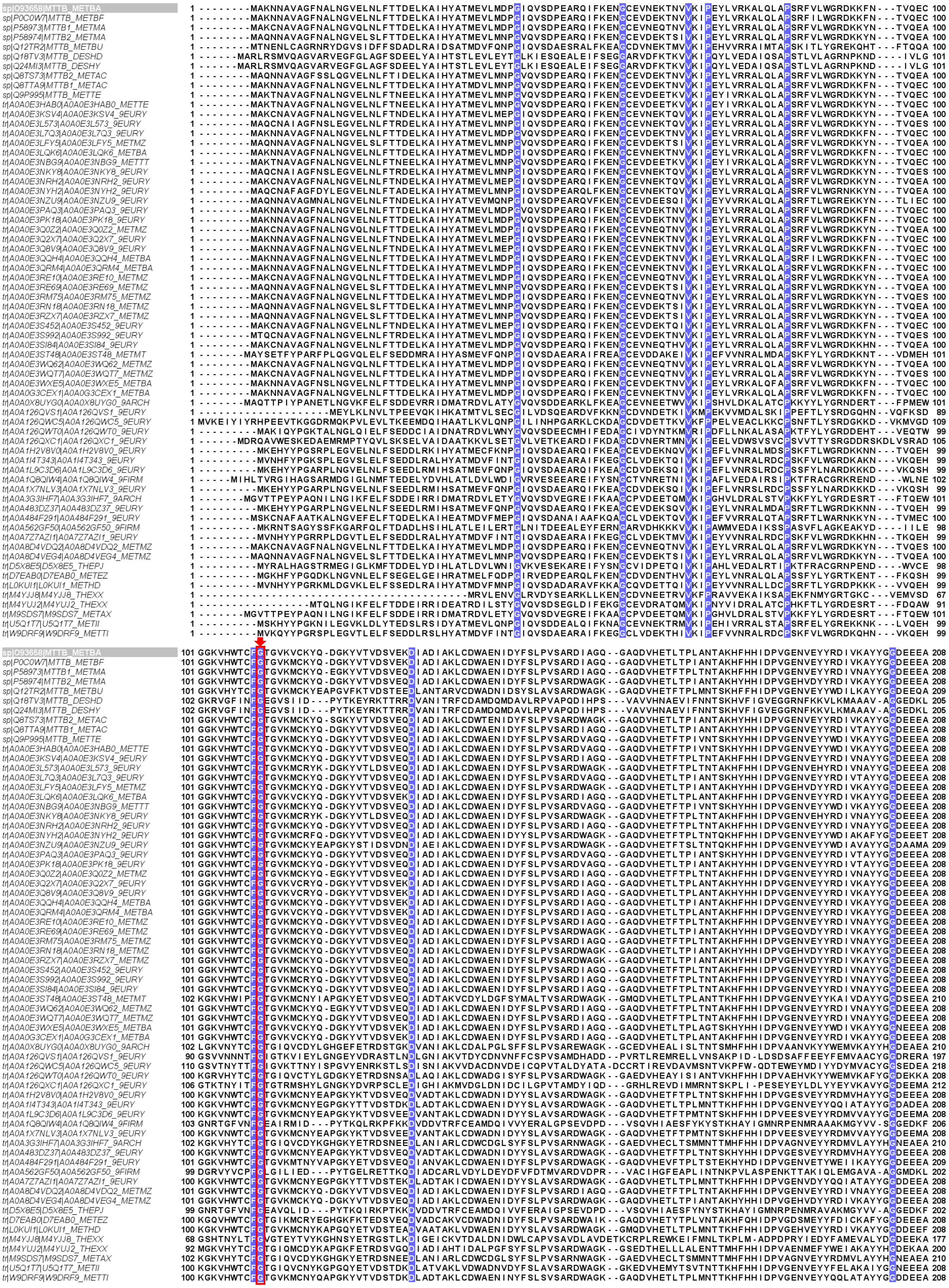

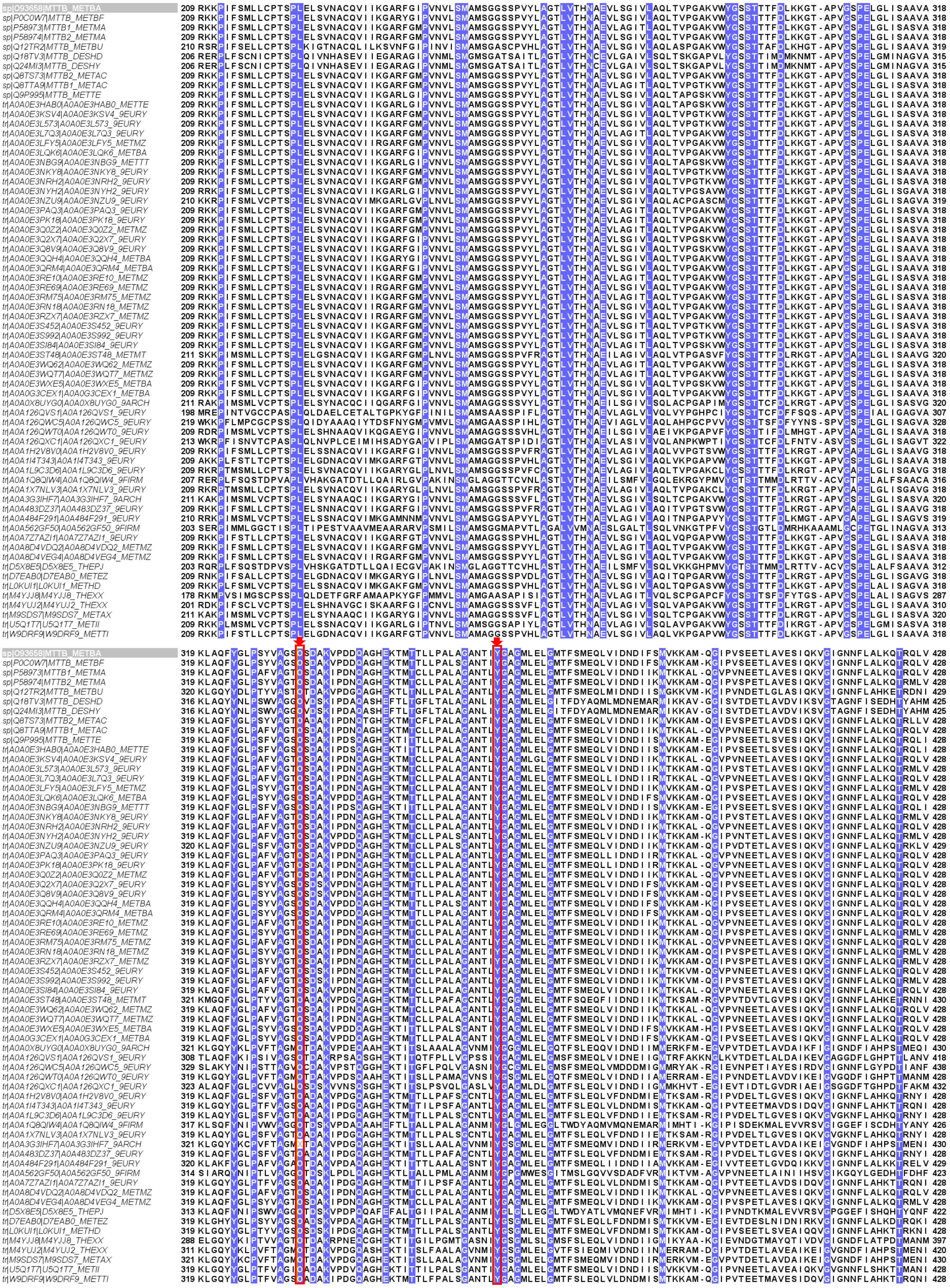

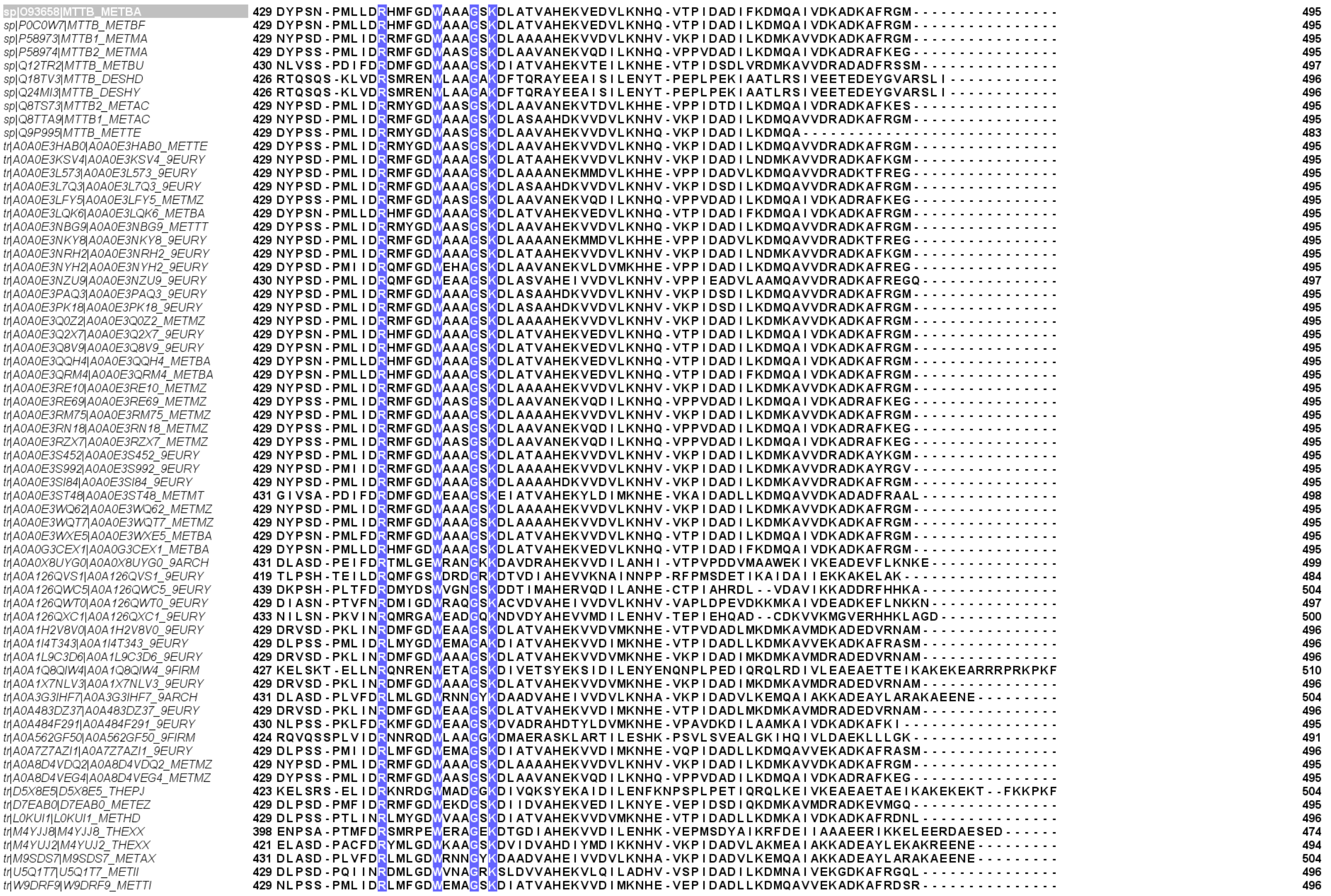

**Supplementary Figure 4. Sequence conservation in pyrrolysine-containing trimethylamine methyltransferases.** The alignment was performed using Clustal Omega and visualized by Jalview. The MttB amino acid sequence (sp|O93658|MTTB_METBA) was used as the reference. Fully conserved residues are shaded in violet while aligned residues important in MttB activation (110G, 334O, and 364Y) are highlighted with red arrows and boxes.

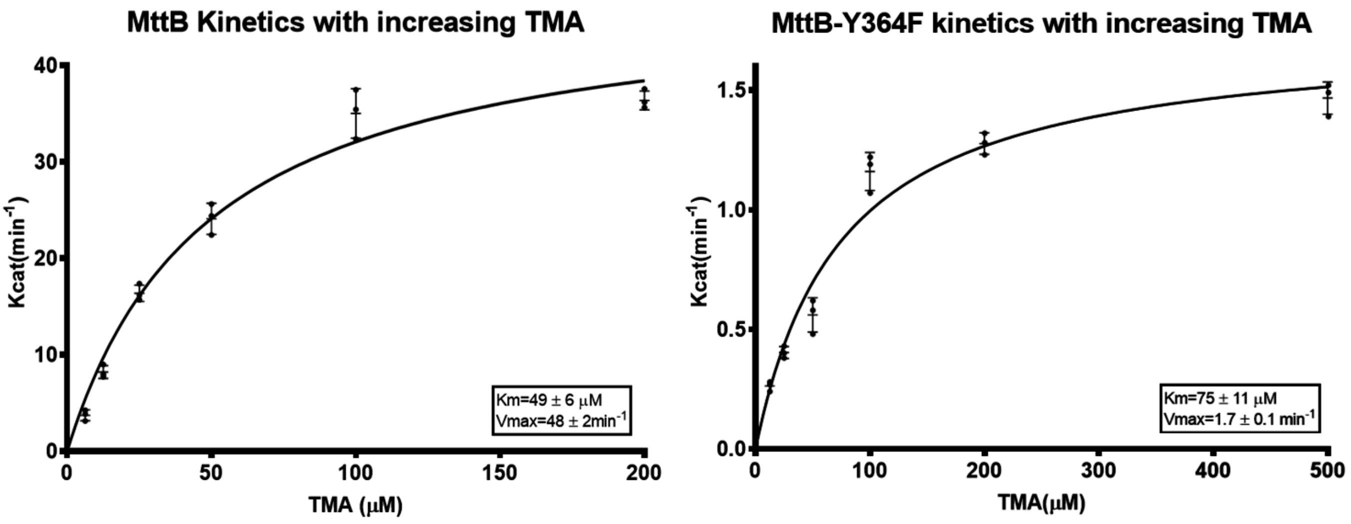


**Supplementary Figure 5. Substitution of the Tyr364 in MttB with phenylalanine results in a less active enzyme with higher $K_M$.** The catalytic functions of the MttB-Y364F variant were analyzed in the same condition with MttB, each with triplicate assays. Data points are plotted individually and are presented with the mean ± SD.

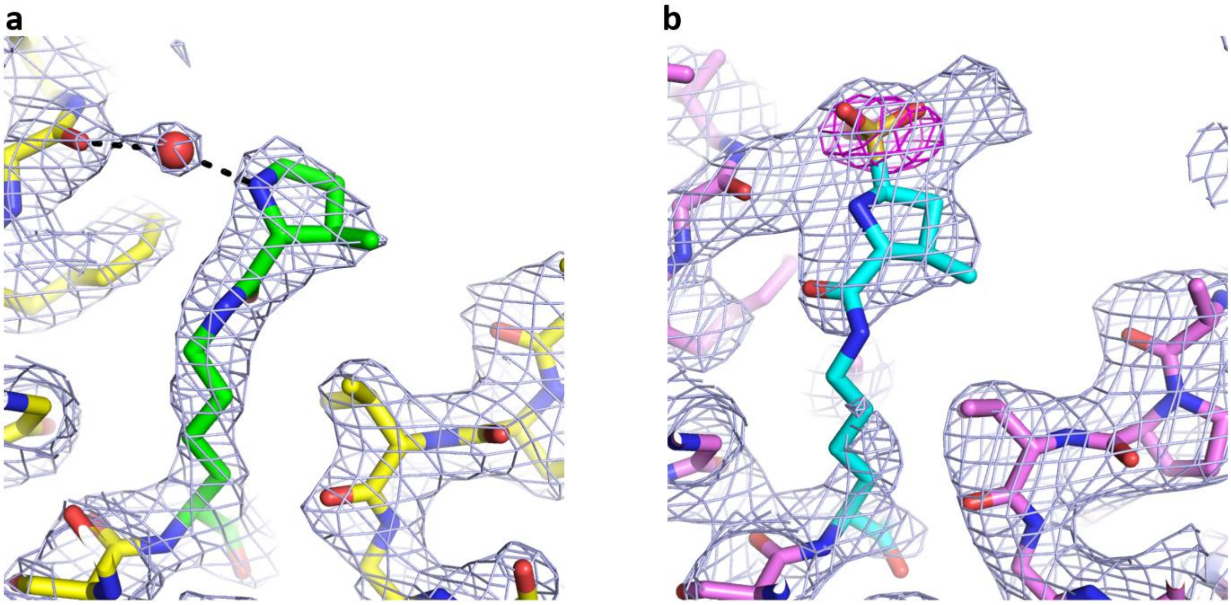


**Supplementary Figure 6. View of the electron density map of residues around pyrrolysine in MttB and sulfite-bound MttB.** (**a**) MttB shown in stick colored in CPK with pyrrolysine carbons in green, the remaining carbons in yellow. The $2F_O$-$F_C$ map displayed at a sigma level of 1.2 (light blue mesh) generated using the program CNS and the hydrogen bonds are shown as dashed black lines. (**b**) Sulfite-bound MttB shown in stick colored in CPK with pyrrolysine carbons in cyan, and remaining carbons in magenta. The $2F_O$-$F_C$ map displayed at a sigma level of 1.0 (light blue mesh) and $F_O$-$F_C$ map displayed at a sigma level of 7.0 (magenta mesh), both generated using the program CNS with sulfite omitted from the model.

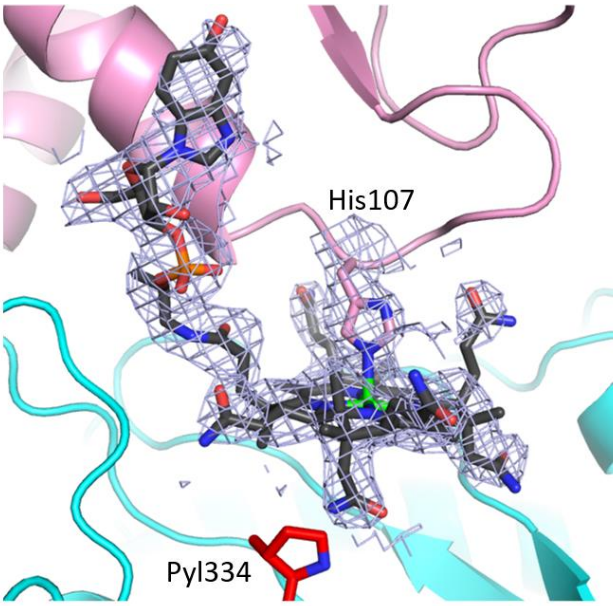


**Supplementary Figure 7. View of the $2F_O$-$F_C$ electron density map of the corrinoid cofactor and its bound histidine in the MttB-MttC structure.** Colors of molecules are same as in Fig. 3d. The $2F_O$-$F_C$ map was generated by CNS and displayed at a sigma level of 1.0 with a 2.5 Å cutoff (light blue mesh).

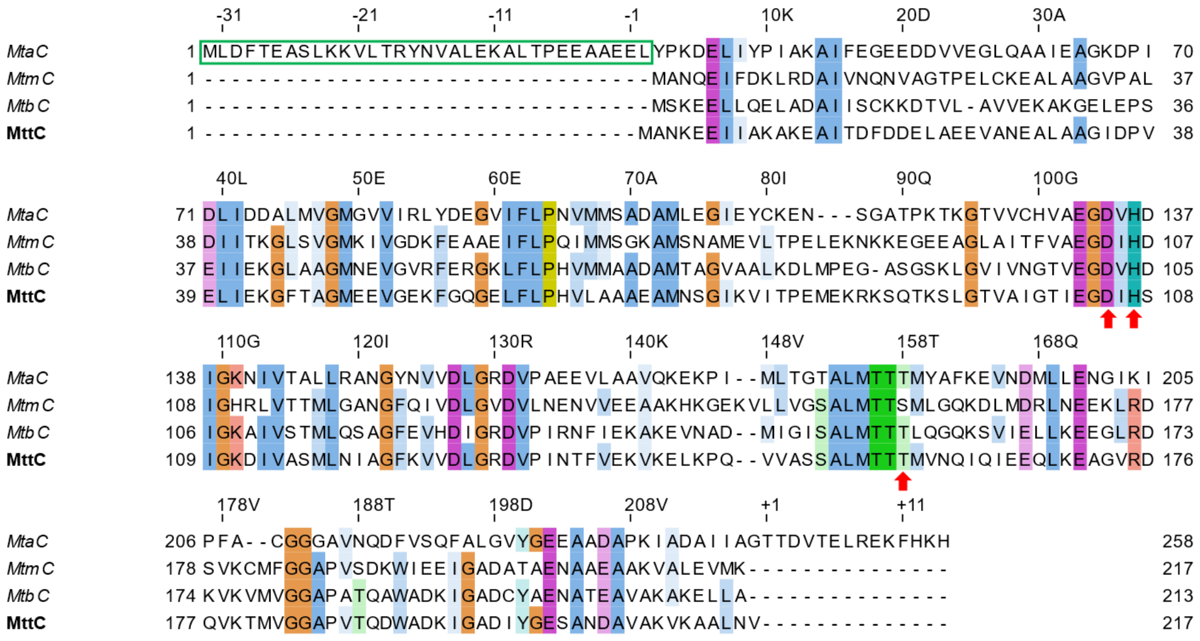


**Supplementary Figure 8. Sequence alignment of corrinoid protein MtmC, MtbC, MttC and MtaC.** The alignment was performed using Clustal Omega and visualized by Jalview. The MttC amino acid sequence was used as the reference. The green box highlights the additional N-terminal region in MtaC, while the red arrows indicate the residues associated with the conserved triad (Asp105-His107-Thr158 in MttC).

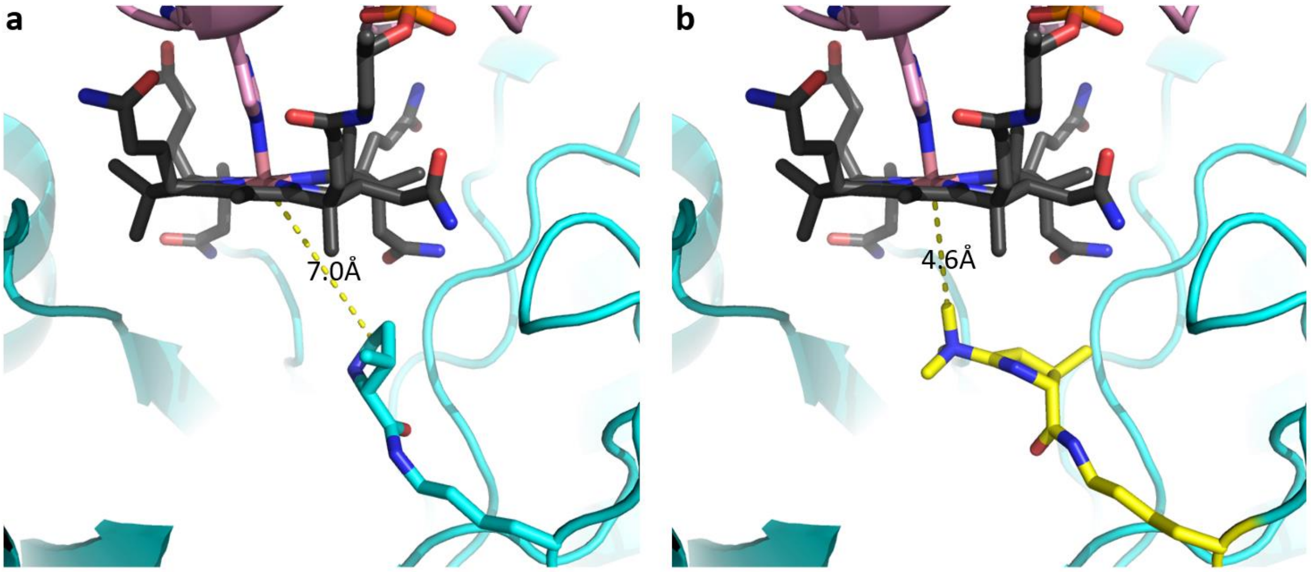


**Supplementary Figure 9. The distance between pyrrolysine and the pentacoordinate His-bound corrinoid cofactor in the MttB-MttC complex is not suitable for the methyl transfer reaction.** View of the active site in (**a**) the structure of the MttB-MttC complex and (**b**) a molecular model of the complex bound to TMA with pyrrolysine oriented so that the TMA is optimally positioned for methyltransfer to the cobalt. In the structure of the MttB-MttC complex, the distance between imine carbon and cobalt in corrinoid cofactor is 7.0 Å, while in the TMA-bound model, the distance between the methyl group to cobalt is at best 4.6 Å. The distance is too long for methyl group transfer to corrinoid cofactor suggesting that the corrinoid could move towards MttB upon its reduction to Co(I).

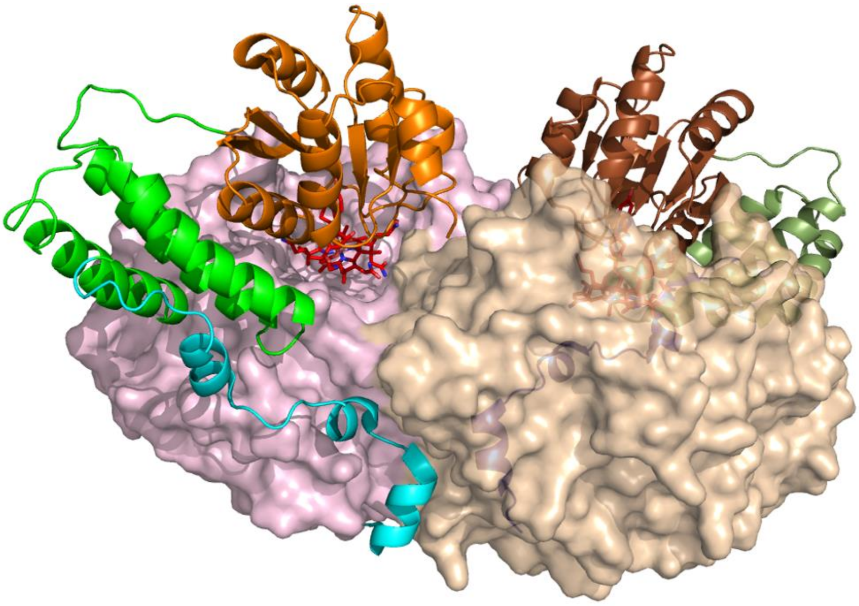

**Supplementary Figure 10. Structure of MtaB-MtaC tetramer (PDB ID: 2I2X).** The two MtaB subunits are shown as surface in light pink/wheat. The cobamide cofactors are shown in red stick and sit in the groove of MtaB. The two MtaC subunits are shown as ribbon with their N-terminal elongated regions (residues 1-39) colored in cyan/blue, cap domains (residues 40-122) in green/olive, and core domains (residues 123-258) in orange/brown.

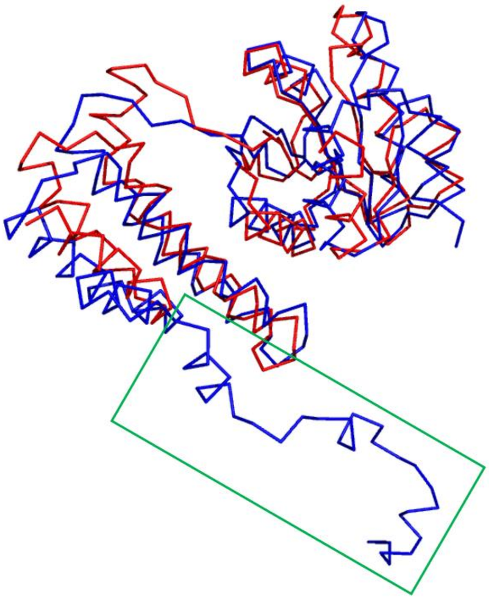

**Supplementary Figure 11. Superposition of $C_\alpha$ traces of MttC and MtaC.** MttC (red) and MtaC (blue) share 37% identity, 56% similarity according to the BLASTP 2.12.0 program. The green box highlights the extra loop in MtaC.
